# Pathophysiology of the gut–eye axis in a Sjögren's disease‐like dry eye mouse model: An exploratory evaluation of dietary heat‐treated *Enterococcus faecalis* FK‐23

**DOI:** 10.1113/EP093816

**Published:** 2026-08-10

**Authors:** Kenji Goto, Shigekazu Takemura, Yukiko Minamiyama, Toshikazu Yoshikawa

**Affiliations:** ^1^ Department of Frontier Life‐science, Graduate School of Medicine Osaka Metropolitan University Abeno‐ku Osaka Japan; ^2^ Research Laboratories Nichinichi Pharmaceutical Co. Ltd Iga‐shi Mie Japan; ^3^ Louis Pasteur Center for Medical Research Sakyo‐ku Kyoto Japan; ^4^ Department of Hepato‐Biliary‐Pancreatic Surgery, Graduate School of Medicine Osaka Metropolitan University Abeno‐ku Osaka Japan; ^5^ Department of Gastroenterology and Hepatology, Graduate School of Medical Science Kyoto Prefectural University of Medicine Kamigyo‐ku, Kyoto‐shi Japan

**Keywords:** dry eye, goblet cell, heat‐treated *Enterococcus faecalis* FK‐23, lacrimal gland, Paneth cell, small intestine

## Abstract

Sjögren's disease is an autoimmune disease characterized by severe dry eye, but its pathophysiology remains unclear. Non‐obese diabetic (NOD) mice develop Sjögren's disease‐like dry eye prior to the onset of diabetes. Although intestinal immune abnormalities precede diabetes in NOD mice, their relationship to dry eye remains poorly understood. This study investigated pathological changes in the lacrimal gland (LG) and small intestine of NOD mice and evaluated the potential effects of dietary heat‐treated *Enterococcus faecalis* FK‐23 (FK‐23). Four‐week‐old male NOD mice were fed either a standard diet or a diet containing 3% FK‐23 for 2 weeks. BALB/c mice served as controls. Tear secretion was measured following pilocarpine administration. LG and small intestine tissues were analysed histologically, and intestinal lymphocytes were evaluated by flow cytometry and next‐generation sequencing. At 4 weeks of age, NOD mice exhibited normal tear secretion and no apparent LG inflammation but showed reduced small intestinal crypt granules and decreased proportions of ILC3 and T helper 17 cells. At 6 weeks, NOD mice demonstrated reduced tear secretion, inflammatory cell infiltration in the LG, and loss of crypt granules and goblet cells in the small intestine. Dietary FK‐23 administration showed trends towards attenuating tear secretion decline, LG inflammation and intestinal pathological changes. Development of LG inflammation and dry eye in NOD mice was associated with intestinal immune and epithelial abnormalities. These findings suggest potential involvement of the gut–eye axis in Sjögren's disease‐like dry eye pathogenesis and indicate that modulation of intestinal homeostasis might represent a therapeutic strategy.

## Abstract 2

Intestinal abnormalities precede Sjögren's disease (SjD)‐like dry eye development in non‐obese diabetic (NOD) mice. Four‐week‐old NOD mice exhibited intestinal epithelial and immune abnormalities before the onset of lacrimal gland inflammation and tear reduction at 6 weeks of age. Dietary heat‐treated *Enterococcus faecalis* FK‐23 showed trends towards suppressing intestinal and lacrimal gland abnormalities. These findings suggest a potential role for the gut–eye axis in SjD‐like dry eye pathogenesis.

## 1 INTRODUCTION

Dry eye cases are increasing because of a variety of factors, including intractable diseases of unknown aetiology, such as Sjögren's disease (SjD), an ageing population, widespread air conditioning, increased use of digital devices such as smartphones and computers, and increased contact lens use. Papas (2021) derived prevalence estimates from a Bayesian posterior distribution by combining a likelihood function generated from all relevant studies reporting dry eye prevalence between 1997 and 2021 with a non‐informative prior distribution. Based on this analysis, the global prevalence of dry eye disease was estimated to be ∼10% (Papas, 2021). Nevertheless, Stapleton et al. (2017) suggested that a substantial proportion of individuals with dry eye disease are unlikely to seek medical care (Stapleton et al., 2017). Autoimmune diseases, such as graft‐versus‐host disease and SjD, can also cause severe dry eye. Various theories have been proposed regarding pathogenesis, but the underlying cause remains poorly understood (Brito‐Zerón et al., 2016). Therefore, SjD treatment is largely symptomatic, and a cure is needed. Owing to the complexity of SjD, its clinical course can be divided into several stages: an initiation stage following endogenous and exogenous factors, chronic inflammation induced by dysregulation of salivary gland epithelial cells and immune system activation, and hyperactivity of B cells (Ogawa et al., 2021). All of these events culminate in the development of dry eye disease owing to destruction of the glandular structure. The transition from innate to adaptive immunity and the involvement of various cell types pose challenges in developing effective treatment strategies for SjD, and the stepwise progression of the disease suggests that earlier intervention is necessary (Wu et al., 2022).

Several SjD animal models exist, including the non‐obese diabetic (NOD) mouse, which spontaneously develops type 1 diabetes. It is used as an SjD‐like dry eye model because the pathology of inflammatory cell infiltration in the lacrimal and salivary glands is similar to that of human SjD. It is more susceptible to dacryoadenitis than other mouse models, and it develops dacryoadenitis before the onset of diabetes (Leiter et al., 1987). The incidence of glandular inflammation in NOD mice is sex‐dependent, with higher rates in the lacrimal glands (LGs) of males and salivary glands of females (Takahashi et al., 1997). It has been reported that inflammatory cell infiltration (dacryoadenitis) in the LG of male NOD mice is absent at 4 weeks but becomes evident from 6–8 weeks (Hunger et al., 1998). T cells predominate in the early stages of infiltration, whereas B cells accumulate rapidly in more advanced stages, eventually becoming predominant (Hunger et al., 1998). In addition, the innate immune system appears to be involved in the development of exocrine gland inflammation (Warner & Núñez, 2013). Innate lymphoid cells (ILCs) have been categorized into three main groups based on their similarity to T helper (Th) cell subsets (Spits et al., 2013). They have been suggested to play important roles in immunity, tissue development and remodelling, and studies have focused on the roles of ILC1, ILC2 and ILC3 in autoimmune and inflammatory diseases (Ebbo et al., 2017). However, there is little evidence demonstrating the involvement of ILCs in dacryoadenitis.

In recent years, the relationship between the intestine and other organs has attracted attention, and eye diseases are no exception. It has been reported that intestinal symptoms appear before the onset of type 1 diabetes in NOD mice (Miranda et al., 2019). Recent reports have confirmed the effectiveness of probiotics in artificially induced dry eye models other than NOD, demonstrating that IRT5, a mixture of five probiotic strains, improves dry eye symptoms (Moon et al., 2020). In addition, *Lactobacillus plantarum* NK151 and *Bifidobacterium bifidum* NK175 have been reported to alleviate dry eye symptoms by regulating tumor necrosis factor‐α, interleukin (IL)‐10 and gut microbiota composition (Yun et al., 2021). Heat‐treated *Enterococcus faecalis* FK‐23 (FK‐23) cells are easily ingested owing to their heat‐treated nature and have been shown to enhance recovery from leucopenia in mice treated with the anticancer drugs 5‐fluorouracil and cyclophosphamide. This study observed an extension of mouse survival and suppression of the decline in antibody‐producing cells in spleen cells (Hasegawa et al., 1994). Furthermore, enzyme‐digested FK‐23 suppressed airway resistance and lung inflammation and increased the expression of regulatory T cells in patients with allergic rhinitis. It also reduced the expression of mast cells in the lung and Th17 cells, which produce IL‐17, in the spleen, resulting in a decrease in cytokines, such as IL‐4 and IL‐6 (Zhang et al., 2012; Zhu et al., 2012). These reports confirm that FK‐23 has the ability to modulate immune responses and might also be effective in treating dry eye disease caused by LG failure. In this study, we examine the differences in intestinal and LG pathology between pre‐onset and onset of SjD‐like dry eye disease mice and investigate whether dietary administration of FK‐23 to non‐onset NOD mice can suppress SjD‐like dry eye symptoms and the immune‐related factors involved.

## 2 MATERIALS AND METHODS

### 2.1 Mice and procedures

Three‐week‐old male non‐obese diabetic/ShiJcl (NOD) mice and BALB/cCrSlc (control; CRL) were purchased from CLEA Japan (Tokyo, Japan). The mice were housed for 1 week in a semi‐barrier animal room (23°C ± 2°C, 50% humidity, 12 h–12 h light–dark cycle and ad libitum feeding). They were divided into six groups consisting of at least five mice per group: 4‐week‐old control (4W_CRL), 4‐week‐old NOD (4W_NOD), 6‐week‐old CRL (6W_CRL), 6‐week‐old CRL+FK‐23 (6W_CRL+FK‐23), 6‐week‐old NOD (6W_NOD) and 6‐week‐old NOD+FK‐23 (6W_NOD+FK‐23). FK‐23 was provided by Nichinichi Pharmaceutical Co., Ltd (Mie, Japan). CRL+FK‐23 and NOD+FK‐23 mice were fed 3% FK‐23 mixed with CE‐2 pellets (CLEA Japan, Tokyo, Japan) ad libitum for 2 weeks, starting at 4 weeks of age. The feeding amounts for each group of 6‐week‐old mice were as follows: 6W_CRL, 3.79 g/day per mouse; 6W_CRL+FK‐23, 3.64 g/day per mouse; 6W_NOD, 3.75 g/day per mouse; and 6W_NOD+FK‐23, 3.74 g/day per mouse. The 6W_CRL+FK‐23 group ingested 109.2 mg/day per mouse of FK‐23, and the 6W_NOD+FK‐23 group ingested 112.2 mg/day per mouse of FK‐23. At 4 or 6 weeks of age, under inhalation anaesthesia with sevoflurane, blood was collected from the hearts of the mice, and tissues were harvested after death by exsanguination. All animal experimental protocols were approved by the Use of Laboratory Animals Committee of Osaka Metropolitan University Graduate School of Medicine (approval no. 23041, 25031). The animal experiments in this study were carried out in accordance with the ARRIVE guidelines and national animal experiment guidelines.

### 2.2 Measurement of tear volumes

Before post‐mortem examination, tear volume was determined by the cotton thread test using a Phenol Red‐impregnated thread (AYUMI Pharmaceutical Corporation, Tokyo, Japan). Mice were anaesthetized by intraperitoneal (i.p.) injection of an anaesthetic agent mixture: 0.3 mg/kg medetomidine (Fujita Pharmaceutical Co., Ltd, Tokyo, Japan), 4.0 mg/kg midazolam (Sandoz K.K., Tokyo, Japan) and 5.0 mg/kg butorphanol (Meiji Animal Health Co., Ltd, Tokyo, Japan) at a volume of 0.1 mL per 10 g of body weight. This mixture was determined based on previous reports (Kawai et al., 2011; Nakamura et al., 2017). After the mice had been fully anaesthetized, 0.5 mg/kg of pilocarpine (FUJIFILM Wako Pure Chemical Corporation, Osaka, Japan) was injected i.p. at a volume of 0.1 mL per 10 g of body weight. The dose was determined based on a previous study measuring tear volume (Ohno et al., 2020). The volume of tear fluid was measured by carefully placing a Phenol Red‐impregnated thread at the canthus of each eye for 30 s before pilocarpine injection and at 2, 7, 12 and 22 min after injection. The length of thread that changed colour owing to absorption of tear fluid was measured (in millimetres). Because tear secretion results are likely to be influenced by individual mouse body weight, tear volume measurements were corrected for body weight. The total tear volume was calculated by summing the length of the colour‐changed thread in each 30 s measurement segment at five measurement points. After tear measurement was completed, 1.5 mg/kg of atipamezole (Fujita Pharmaceutical Co., Ltd) was injected i.p. at a volume of 0.1 mL per 10 g of body weight, and the mice awoke immediately.

### 2.3 Histological analysis

Small intestine (middle portion of the jejunum) and LG samples were isolated, fixed with 10% neutral buffered formalin and embedded in paraffin. For each animal, five sections (5 µm thick) were cut from the tissue samples. Tissue sample sections were stained with Haematoxylin and Eosin. Jejunum samples were stained with Alcian Blue and Nuclear Fast Red to determine the expression of goblet cells. Images of tissue sections were obtained using a BZ‐50 microscope (Olympus Corporation, Tokyo, Japan) and a DS‐Ri2 digital camera (Nikon Corporation, Tokyo, Japan) and processed using ImageJ software (National Institutes of Health, Bethesda, MD, USA). The degree of LG inflammation was expressed as a focus score, defined as the percentage of the infiltrated area relative to the total area of the LG tissue section. Image analysis was performed using ImageJ software in combination with manual evaluation, whereby areas of infiltration were carefully identified and delineated by the investigator. The results were expressed as the mean ± SD, calculated from the mean values of per animal. For analysis of crypt granules and goblet cells, the jejunum was evaluated using ImageJ software in combination with manual evaluation. Specifically, goblet cells were defined as cells that stained clearly blue with Alcian Blue staining. Goblet cells in villi and crypts were counted and expressed as the number per unit area of jejunal tissue. Granules were defined as red, round, granular structures observed within the crypts. These were counted in five crypts and expressed as the number of granules per crypt.

### 2.4 Cell isolation from small intestinal lamina propria

Small intestinal lamina propria (SILP) mononuclear cells were isolated from the small intestine of 4‐ or 6‐week‐old male NOD mice and healthy CRL mice (Saksida et al., 2023). Briefly, mice were killed, and the small intestine from below the duodenum to the caecum was removed and cut into lengths of ∼5 cm. After removal of the intestinal contents and Peyer's patches, the tissue pieces were opened longitudinally, further cut into ∼1 cm lengths, and thoroughly washed three times with cold Hanks’ balanced salt solution (HBSS). After this, the samples were washed in HBSS containing 2% fetal calf serum (FCS; BioWest, Nuaillé, France) and 2.5 mM dithiothreitol (DTT; FUJIFILM Wako Pure Chemical Corporation) in an orbital shaker (250 rpm) for 15 min to reduce the presence of mucus. The samples were then washed three times in HBSS containing 2% FCS and 5 mM EDTA (250 rpm, 15 min) to remove intestinal epithelial cells. The tissue pieces were finally collected, washed in Roswell Park Memorial Institute (RPMI) medium (Shimadzu Diagnostics Corporation, Tokyo, Japan) supplemented with 10% FCS (250 rpm, 10 min) and cut into small pieces with surgical scissors. The finely cut sample was resuspended in RPMI 10% FCS with Collagenase D (700 µg/mL) and DNase I (0.1 mg/mL) (both from Roche Diagnostics GmbH, Mannheim, Germany) and incubated for 1 h at 37°C in an orbital shaker (350 rpm). After digestion, the tissue sample was meshed using a 70 µm cell strainer (Becton Dickinson and Company, NJ, USA), and the collected solution was centrifuged at 550*g* for 5 min. The pellet was resuspended in a Percoll (Global Life Sciences Technologies Japan, Tokyo, Japan) gradient solution (40%), then layered upon 80% Percoll, after which it was centrifuged at 400 rpm for 20 min without rotor brakes. SILP lymphocytes (found at the interface between 40% and 80% Percoll) were collected, washed twice in HBSS, and resuspended in HBSS for further analysis.

### 2.5 Cell isolation from the lacrimal gland

Single cells were isolated from the LG using the method of Li et al. (2022) and Seo et al. (2014), with modifications. Single‐cell suspensions from one side of the LG from 4‐ or 6‐week‐old male NOD mice and healthy CRL mice in each condition were prepared by treating minced tissue fragments with 2 mg/mL collagenase D and 15 µg/mL DNase I for 1 h at 37°C. After digestion, the tissue was meshed using a 70 µm cell strainer and centrifuged at 550*g* for 5 min. The pellet was washed twice in HBSS and resuspended in HBSS for further analysis.

### 2.6 Flow cytometry

For flow cytometry analysis, 1.0–3.0 × 10^5^ cells were washed and reacted with the respective antibodies. For blocking Fc receptors in flow cytometric analysis, cells were pre‐incubated with TruStain FcX™ PLUS (RRID: AB_2783137; catalogue no. 156603; BioLegend, CA, USA) at 1.0 µg per 10^6^ cells in a volume of 100 µL for 10 min on ice prior to immunostaining. Cells were washed with HBSS and surface antigen stained with some antibodies (Table 1) for 20 min on ice. ILCs and Th17 cells were stained according to each detection panel (Table 2). After antibody staining, the cells were washed twice with HBSS, then suspended in 500 µL of HBSS. They were passed through the mesh of a 5 mL polystyrene round‐bottomed tube with a cell strainer snap cap (Corning Life Sciences Co., Ltd, AZ, USA) to prepare a sample. Immediately before flow cytometry analysis, the cells were incubated with 5 µL of 7‐amino‐actinomycin D (RRID: N/A; catalogue no. 420403; BioLegend) for 5 min on ice, then quantified by flow cytometry analysis using X‐20 (Becton Dickinson and Company).

**TABLE 1:** Antibodies.

Antibody	Company name	RRID	Catalogue no.	Concentration (per 10^6^ cells/100 µL)
Brilliant Violet 421™ anti‐mouse CD194 (CCR4)	Biolegend, CA, USA	AB_2650890	131218	0.5 µg
Brilliant Violet 711™ anti‐mouse CD4	Biolegend, CA, USA	AB_2562607	100557	0.5 µg
FITC anti‐mouse F4/80	Biolegend, CA, USA	AB_893500	123107	0.25 µg
FITC anti‐mouse CD5	Biolegend, CA, USA	AB_312734	100605	1.0 µg
FITC anti‐mouse Ly‐6G/Ly‐6C (Gr‐1)	Biolegend, CA, USA	AB_313371	108406	0.25 µg
PE anti‐mouse CD183 (CXCR3)	Biolegend, CA, USA	AB_1027656	126505	0.25 µg
PE/Cyanine7 anti‐mouse CD196 (CCR6)	Biolegend, CA, USA	AB_2072798	129816	1.0 µg
APC anti‐mouse LPAM‐1 (Integrin α4β7)	Biolegend, CA, USA	AB_10719833	120607	0.25 µg
APC/Cyanine7 anti‐mouse CD3	Biolegend, CA, USA	AB_2057374	100221	0.25 µg
Brilliant Violet 421™ anti‐mouse CD90.2 (Thy1.2)	Biolegend, CA, USA	AB_2632888	105341	0.25 µg
Brilliant Violet 510™ anti‐mouse CD45	Biolegend, CA, USA	AB_2563061	103138	0.5 µg
FITC anti‐mouse CD3	Biolegend, CA, USA	AB_312660	100203	1.0 µg
APC anti‐mouse CD49b	Biolegend, CA, USA	AB_2566100	103515	0.125 µg
TER‐119 Monoclonal, FITC, eBioscience™	Thermo Fisher Scientific, Tokyo, Japan	AB_465310	11592181	0.25 µg
CD11b Monoclonal, Alexa Fluor™ 488, eBioscience™	Thermo Fisher Scientific, Tokyo, Japan	AB_469901	53011282	0.5 µg
ST2 Polyclonal Antibody, PE Conjugated	Bioss, MA, USA	AB_11058616	bs‐2382R‐PE‐Cy5.5	2.0 µg
Alexa Fluor^®^ 700 Rat Anti‐Mouse CD335 (NKp46)	Becton Dickinson and Company, NJ, USA	AB_10561840	561169	0.4 µg

**TABLE 2:** Antibody staining panel for each cell type.

Innate lymphoid cell detection panel	T helper 17 cell detection panel
Brilliant Violet 421™ anti‐mouse CD90.2 (Thy1.2)	Brilliant Violet 421™ anti‐mouse CD194 (CCR4)
Brilliant Violet 510™ anti‐mouse CD45	Brilliant Violet 711™ anti‐mouse CD4
FITC anti‐mouse CD5	FITC anti‐mouse F4/80
FITC anti‐mouse CD3	FITC anti‐mouse CD5
FITC anti‐mouse Ly‐6G/Ly‐6C (Gr‐1)	FITC anti‐mouse Ly‐6G/Ly‐6C (Gr‐1)
TER‐119 Monoclonal, FITC, eBioscience™	TER‐119 Monoclonal, FITC, eBioscience™
CD11b Monoclonal, Alexa Fluor™ 488, eBioscience™	PE anti‐mouse CD183 (CXCR3)
ST2 Polyclonal Antibody, PE Conjugated	PE/Cyanine7 anti‐mouse CD196 (CCR6)
Alexa Fluor^®^ 700 Rat Anti‐Mouse CD335 (NKp46)	APC anti‐mouse LPAM‐1 (Integrin α4β7)
	APC/Cyanine7 anti‐mouse CD3

### 2.7 Transcriptome analysis

Total RNA was isolated from the SILP cells using Sepasol^®^‐RNA I Super G (NACALAI TESQUE, Kyoto, Japan). RNA sequencing and analysis were outsourced to Novogene Bioinformatics Technology Co., Ltd (Beijing, China). Messenger RNA was purified from total RNA using poly‐T oligo‐attached magnetic beads. After fragmentation, first‐strand complementary DNA was synthesized using random hexamer primers. Then, second‐strand complementary DNA was synthesized using dUTP instead of dTTP. The directional library was ready after end repair, A‐tailing, adapter ligation, size selection, USER enzyme digestion, amplification and purification. The library was checked using Qubit and real‐time PCR for quantification and a bioanalyser for detection of size distribution. After library quality control, different libraries were pooled based on the effective concentration and targeted data amount, then subjected to Illumina sequencing. The basic principle of sequencing is ‘sequencing by synthesis’, whereby fluorescently labelled dNTPs, DNA polymerase and adapter primers are added to the sequencing flow cell for amplification. As each sequencing cluster extends its complementary strand, the addition of each fluorescently labelled dNTP releases a corresponding fluorescence signal. The sequencer captures these fluorescence signals and converts them into sequencing peaks through computer software, thereby obtaining the sequence information of the target fragment. Raw data (raw reads) in FASTQ format were processed initially using fastp software. In this step, clean data (clean reads) were obtained by removing reads containing adapter, reads containing poly‐N and low‐quality reads from raw data. At the same time, the Q20, Q30 and GC content of the clean data were calculated. All the downstream analyses were based on clean, high‐quality data. Reference genome and gene model annotation files were downloaded from the genome website. HISAT2 (v.2.2.1) was used to build the index of the reference genome and to align paired‐end clean reads to the reference genome. HISAT2 can use gene model annotation files to create splice‐aware alignments, providing better alignment accuracy than other, non‐splice alignment tools. featureCounts (v.2.0.6) was used to count the read numbers mapped to each gene. Then, the expected number of fragments per kilobase of transcript sequence per million base pairs sequenced (FPKM) of each gene was calculated based on the length of the gene and the read count mapped to it. FPKM considers the effect of sequencing depth and gene length for the reads count at the same time and is commonly used to estimate gene expression levels. For DESeq2 with biological replicates, differential expression analysis for two conditions/groups was performed using the DESeq2 R package (v.1.42.0). DESeq2 provides statistical programs for determining differential expression in digital gene expression data using models based on a negative binomial distribution. The resulting *P*‐values were adjusted using Benjamini and Hochberg's methods to control the error discovery rate. The threshold of significant differential expression was as follows: *P*_adj_ ≤ 0.05 and |log2(foldchange)| ≥ 1. For edgeR without biological replicates, prior to differential gene expression analysis, for each sequencing library, read counts were adjusted using the *edgeR* R package (v.4.0.16) by scaling normalization factors to eliminate differences in sequencing depth between samples, followed by differential expression analysis. The resulting *P*‐value was adjusted using Benjamini and Hochberg's methods to control the error discovery rate. The threshold for significant differential expression was as follows: *P*_adj_ ≤ 0.05 and |log2(foldchange)| ≥ 1. If the number of different genes screened according to the above threshold was too small (<100), there would probably be no significant results in the subsequent functional enrichment analysis. Therefore, we reduced the threshold appropriately for screening different genes according to the specific conditions of the project. The adjusted threshold for samples with biological replicates was DESeq2 *P*‐value ≤ 0.05 and |log2FoldChange| ≥ 0.0. The adjusted threshold for samples without biological was edgeR *P*‐value ≤ 0.05 and |log2FoldChange| ≥ 1.0.

### 2.8 Statistical analysis

Data are presented as the mean ± SD. Statistical analysis was performed using Student's unpaired *t*‐test or ANOVA, followed by Tukey's *post hoc* multiple comparison test. Values of *P* < 0.05 were considered statistically significant. All experiments, excluding RNA sequencing, were conducted three times using independent experimental systems.

## 3 RESULTS

### 3.1 Pathology of 4‐week‐old NOD mice

#### 3.1.1 Tear volume and lacrimal gland

**FIGURE 1:**
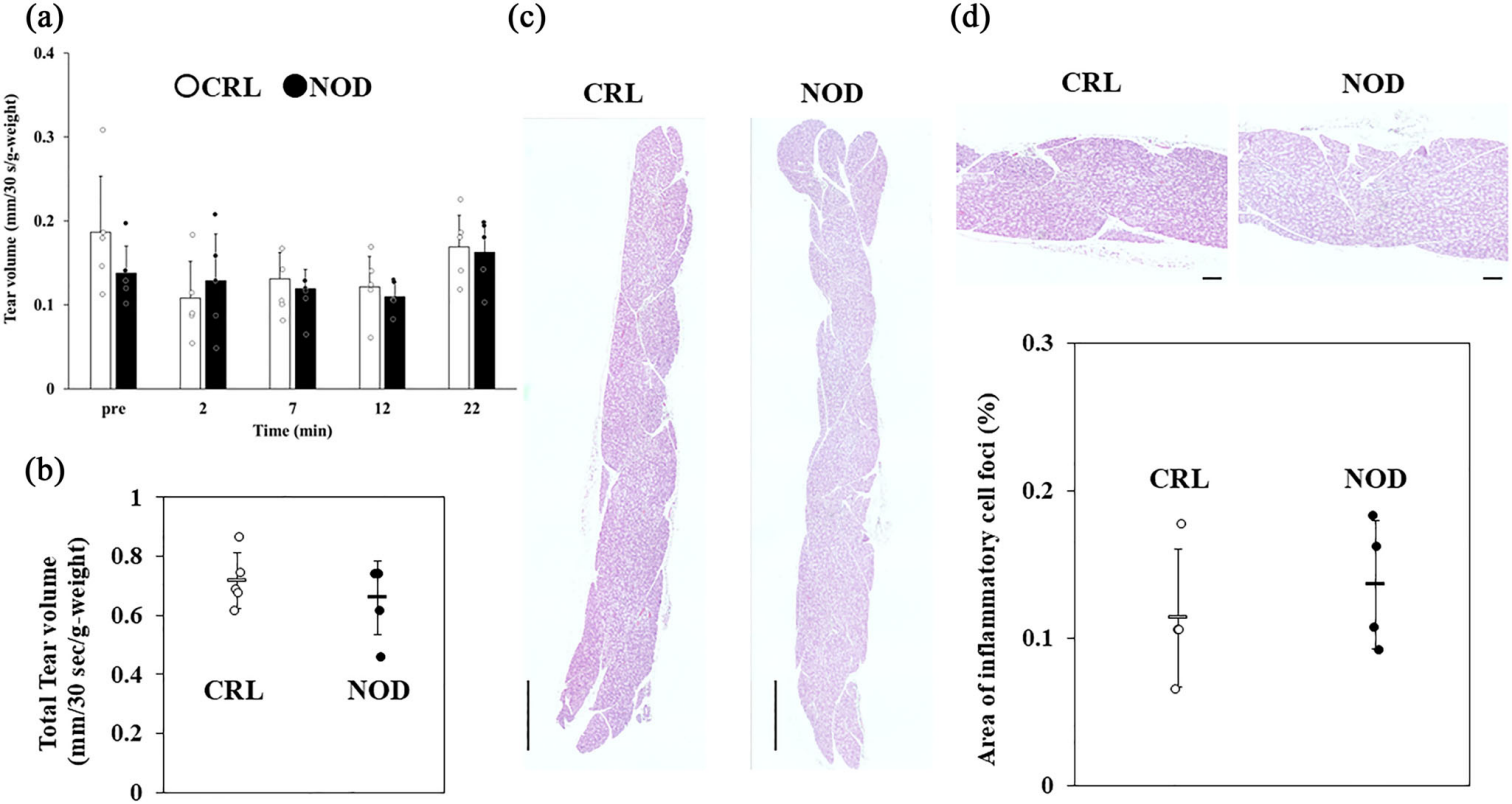
Lacrimal gland symptoms in 4‐week‐old NOD mice. (a) Experimental design for the measurement of tear secretion. We measured the length of the colour‐changed Phenol Red‐impregnated thread to estimate tear volume. Tear volume was measured for 30 s before pilocarpine injection and at 2, 7, 12 and 22 min after injection. (b) Total tear volume after pilocarpine treatment in male CRL and NOD mice at 4 weeks of age. (c) Representative cross‐sections of Haematoxylin‐ and Eosin‐stained lacrimal glands from male CRL and NOD mice at 4 weeks of age. Scale bars: 500 µm. (d) Area of inflammatory cell foci in the lacrimal gland. Scale bars: 100 µm. The obtained values are presented as the mean ± SD vs. the BALB/c group. All experiments were conducted with four or five animals in each group. Abbreviations: CRL, control; NOD, non‐obese diabetic.

Initially, we measured tear volume and confirmed the pathology of the LG at 4 weeks of age. The timing of each measurement and the total tear volume of 4‐week‐old NOD mice were similar to those of CRL mice (Figure 1a,b). The LG was stained with Haematoxylin and Eosin, then observed under a microscope. Inflammatory cells did not infiltrate the LG of 4‐week‐old NOD mice (Figure 1c,d).

#### 3.1.2 Small intestine

**FIGURE 2:**
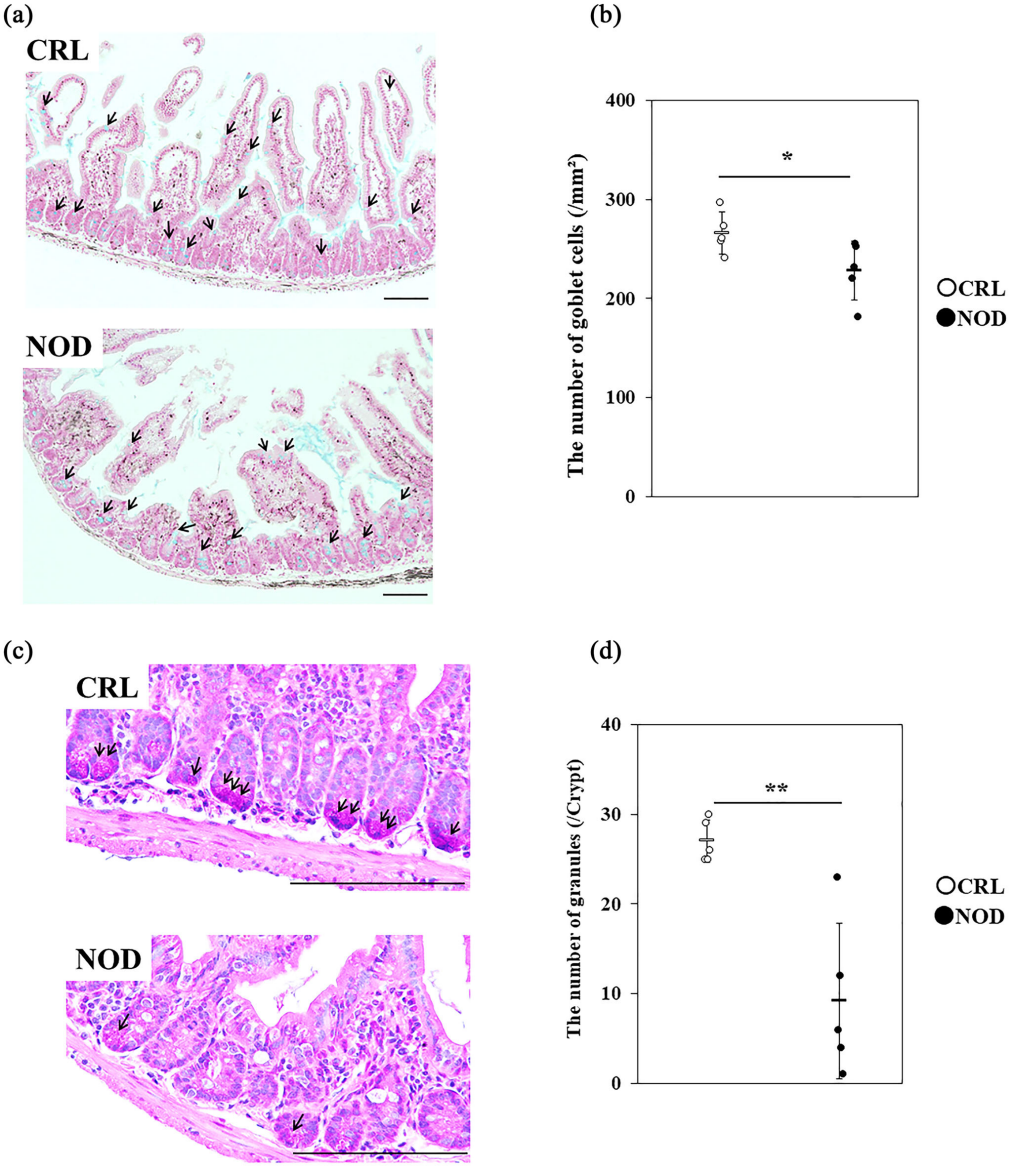
Small intestinal signs in 4‐week‐old NOD mice. (a) Alcian Blue staining of small intestine specimens. Scale bars: 100 µm. Arrows show examples of goblet cells. (b) Number of goblet cells in small intestinal specimens from CRL and NOD mice. (c) Haematoxylin and Eosin staining of small intestine specimens. Scale bars: 100 µm. Arrows show examples of granules. (d) Number of granules in crypts of small intestinal specimens from CRL and NOD mice. The obtained values are presented as the mean ± SD; ^*^ *P* < 0.05 and ^**^ *P* < 0.01 vs. CRL group. All experiments were conducted with five animals in each group. Abbreviations: CRL, control; NOD, non‐obese diabetic.

We investigated the pathology that occurred in the small intestine at 4 weeks of age. Alcian Blue staining revealed that goblet cell expression was lower in NOD mice than in CRL mice (Figure 2a,b). Haematoxylin and Eosin staining revealed a lower expression of granules in the small intestinal crypts (Figure 2c,d).

#### 3.1.3 Fluorescence‐activated cell sorting

To confirm the expression of immune cells, we performed flow cytometry analysis (fluorescence‐activated cell sorting; FACS) of SILP and LG. Here, we confirmed the expression of innate ILCs and Th17 cells and demonstrated this strategy (Figure 3a,b). Lower expression of CD90.2^+^ cells and ILC3s in the small intestine of NOD mice was observed by flow cytometry analysis. In contrast, ILC1 expression was higher. The expression of CD90.2^−^ cells was higher than in CRL mice, with ILC2 being more abundant (Figure 3c). Th17 cells were less abundant in the small intestine of NOD mice than in CRL mice (Figure 3d). In the LG, CD90.2^+^ cells, ILC1 and ILC3 did not differ between CRL and NOD mice, but CD90.2^−^ cells and ILC2 were increased. However, ILCreg expression was lower in NOD mice. NOD mice also had more natural killer (NK) cells than CRL mice. (Figure 3e). Furthermore, Th17 cells were more abundant in the LG of NOD mice than in that of CRL mice, and the expression of the gut‐homing factor α4β7 integrin was reduced (Figure 3f). [Correction added on 21 August 2026, after first online publication: The wording “less abundant” has been changed to “more abundant” in this sentence.]

FIGURE 3Flow cytometry analysis of small intestinal lamina propria and lacrimal glands of 4‐week‐old NOD mice. (a) FACS strategy for ILCs. (b) FACS strategy for Th17 cells. (c) ILCs in the SILP. (d) Th17 cells in the SILP. (e) ILCs in the LG. (f) Th17 in the LG. The obtained values are presented as the mean ± SD; ^**^ *P* < 0.01 vs. CRL group. All experiments were conducted with five animals in each group. Abbreviations: CRL, control; FACS, fluorescence‐activated cell sorting; ILCs, innate lymphoid cells; LG, lacrimal gland; NOD, non‐obese diabetic; SILP, small intestinal lamina propria; Th17, T helper 17.

**Figure:**
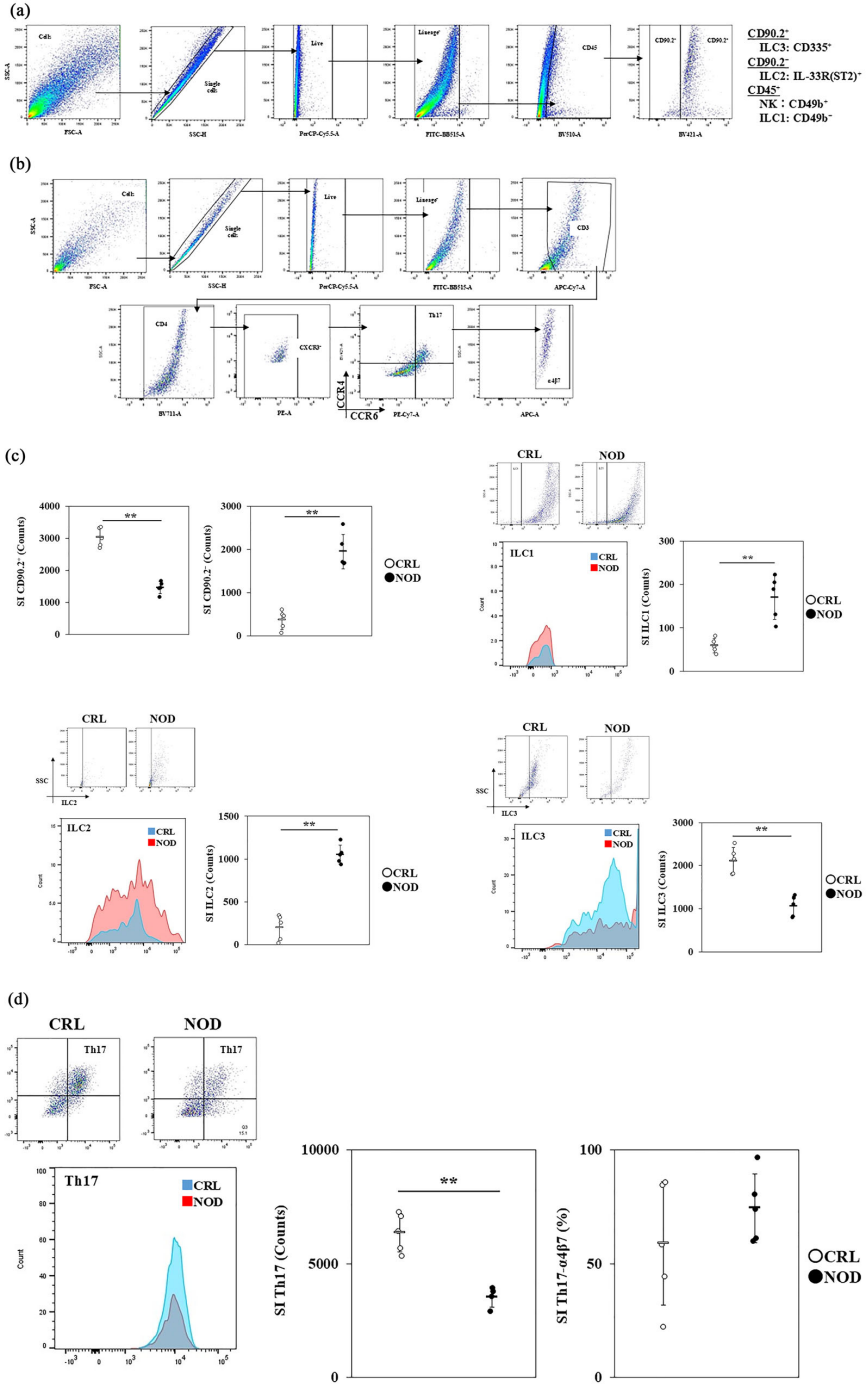

**Figure:**
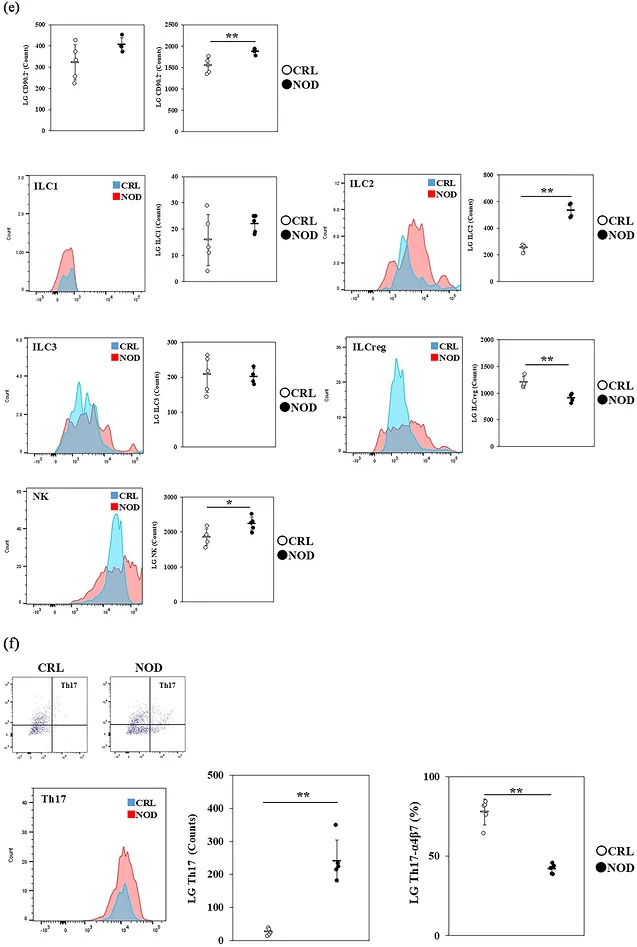

### 3.2 Transcriptome analysis

**FIGURE 4:**
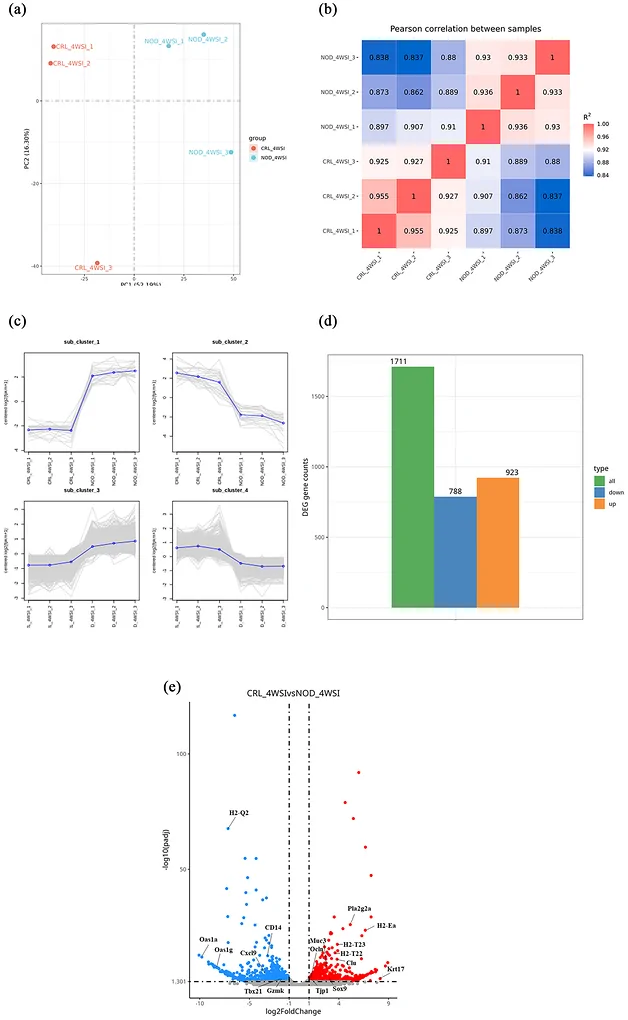
Next‐generation sequencing of small intestinal lymphocytes from 4‐week‐old NOD mice. (a) Two‐dimensional principal component analysis. (b) Pearson correlation. (c) Subcluster. (d) Differentially expressed gene counts. (e) Volcano plot. Abbreviations: CRL, control; NOD, non‐obese diabetic.

To assess the expression of genes comprehensively, we performed RNA sequencing of the small intestinal lymphocytes of 4‐week‐old NOD and BALB/c mice. Multidimensional scaling analysis and Pearson correlation revealed that the gene expression patterns in small intestinal lymphocytes of NOD mice were different at 4 weeks of age (Figure 4a–c). Differentially expressed gene analysis revealed 1711 genes that satisfied a fold change of either ≥2 or ≤1/2, with an independent *t*‐test *P*‐value of <0.05 in at least one comparison pair (Figure 4d). A total of 788 genes showed decreased expression in the CRL mice compared with the NOD mice, whereas 923 genes showed increased expression in the CRL mice compared with the NOD mice. Immune‐related genes are shown in a volcano plot in Figure 4e. The results showed that the NOD mice had increased *H2‐Q2*, a gene involved in cellular stress monitoring and interaction with specific T cells/NK cells, and interferon (IFN)‐inducible enzymes (*Oas1a* and *Oas1g*) compared with CRL mice. In contrast, NOD mice showed low expression of genes involved in bacterial membrane degradation (*PLA2g2a*), regulation of granule secretion (clusterin), Paneth cell differentiation and stem cell maintenance (*Sox9*), MHC class II activation of CD4^+^ T cells (*H2‐Eα*), and suppression of NK cells and CD8^+^ regulatory T cells (*H2‐T23* and *H2‐T22*). To a lesser extent, the expression in NOD mice of genes involved in TJs and hypoxic stress (*Ocln*) and epithelial formation (*Cldn4*) was also decreased.

### 3.3 Pathology of 6‐week‐old NOD mice

#### 3.3.1 Tear volume and lacrimal gland

**FIGURE 5:**
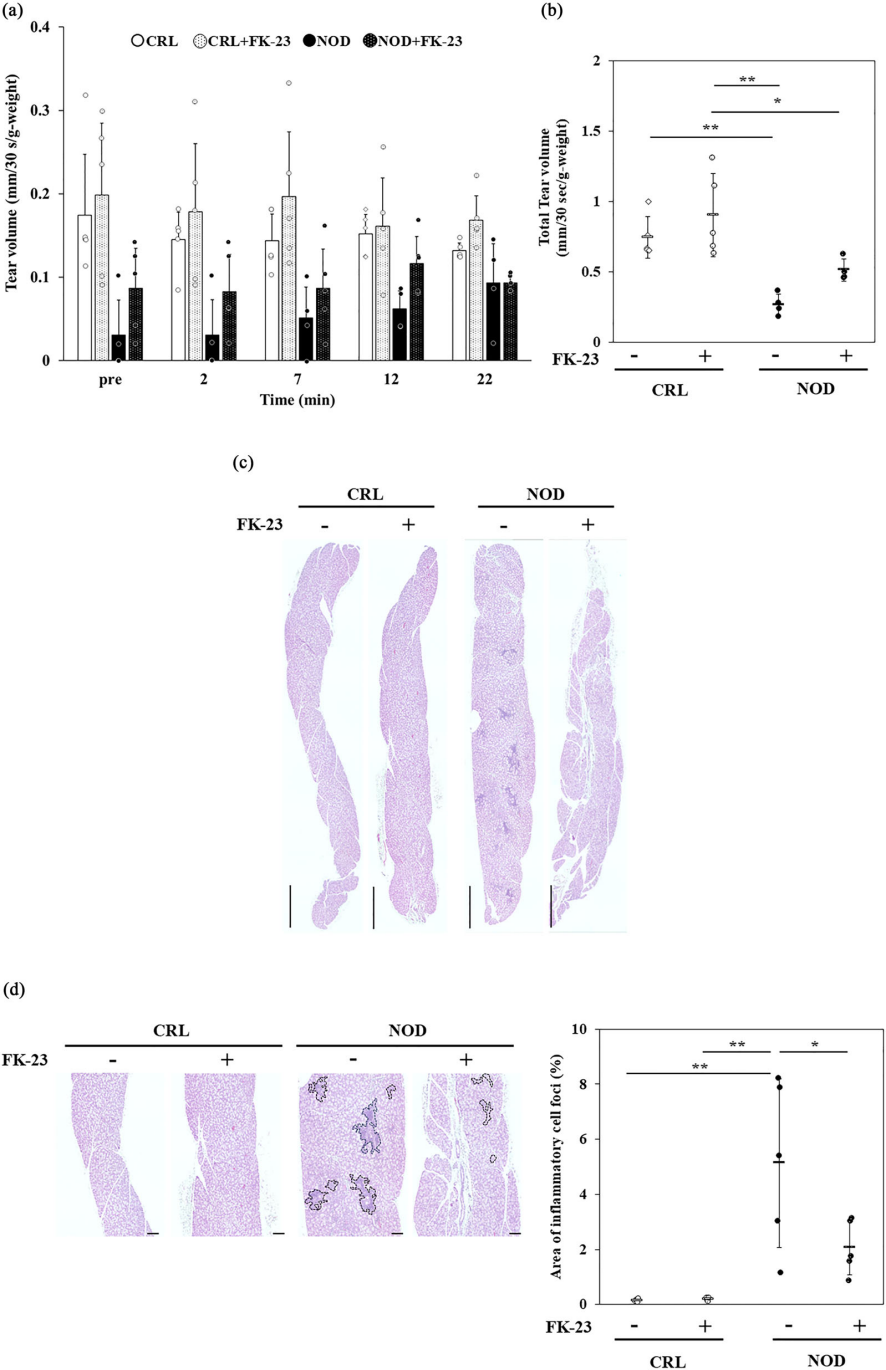
Lacrimal gland symptoms in 6‐week‐old NOD mice and the influence of FK‐23. (a) Experimental design for the measurement of tear secretion. We measured the length of the colour‐changed Phenol Red‐impregnated thread to estimate tear volume. Tear volume was measured for 30 s before injection of pilocarpine and at 2, 7, 12 and 22 min after injection. (b) Total tear volume after pilocarpine treatment in male CRL and NOD mice at 6 weeks of age. (c) Representative cross‐sections of Haematoxylin‐ and Eosin‐stained lacrimal glands from male CRL and NOD mice at 6 weeks of age. Scale bars: 500 µm. (d) Area of inflammatory cell foci in the lacrimal gland. Scale bars: 100 µm. The dotted box shows infiltration of inflammatory cells. Values are presented as the mean ± SD. Tukey's *post hoc* test was used for comparisons among groups (*n* = 4 or 5). ^*^ *P* < 0.05 and ^**^ *P* < 0.01. Abbreviations: CRL, control; FK‐23, *Enterococcus faecalis* FK‐23; NOD, non‐obese diabetic.

We observed the pathology at 6 weeks of age, when LG symptoms began to appear, and examined the effects of dietary FK‐23 on the mice. Compared with the CRL mice, the 6‐week‐old NOD mice showed a tendency towards decreased tear volume at each measurement point, and the total tear volume was also decreased; however, this symptom was suppressed in NOD mice fed FK‐23 (Figure 5a,b). In 6‐week‐old NOD mice, inflammatory cells infiltrated the LG, but this symptom was suppressed in NOD mice fed FK‐23 (Figure 5c,d).

#### 3.3.2 Small intestine

**FIGURE 6:**
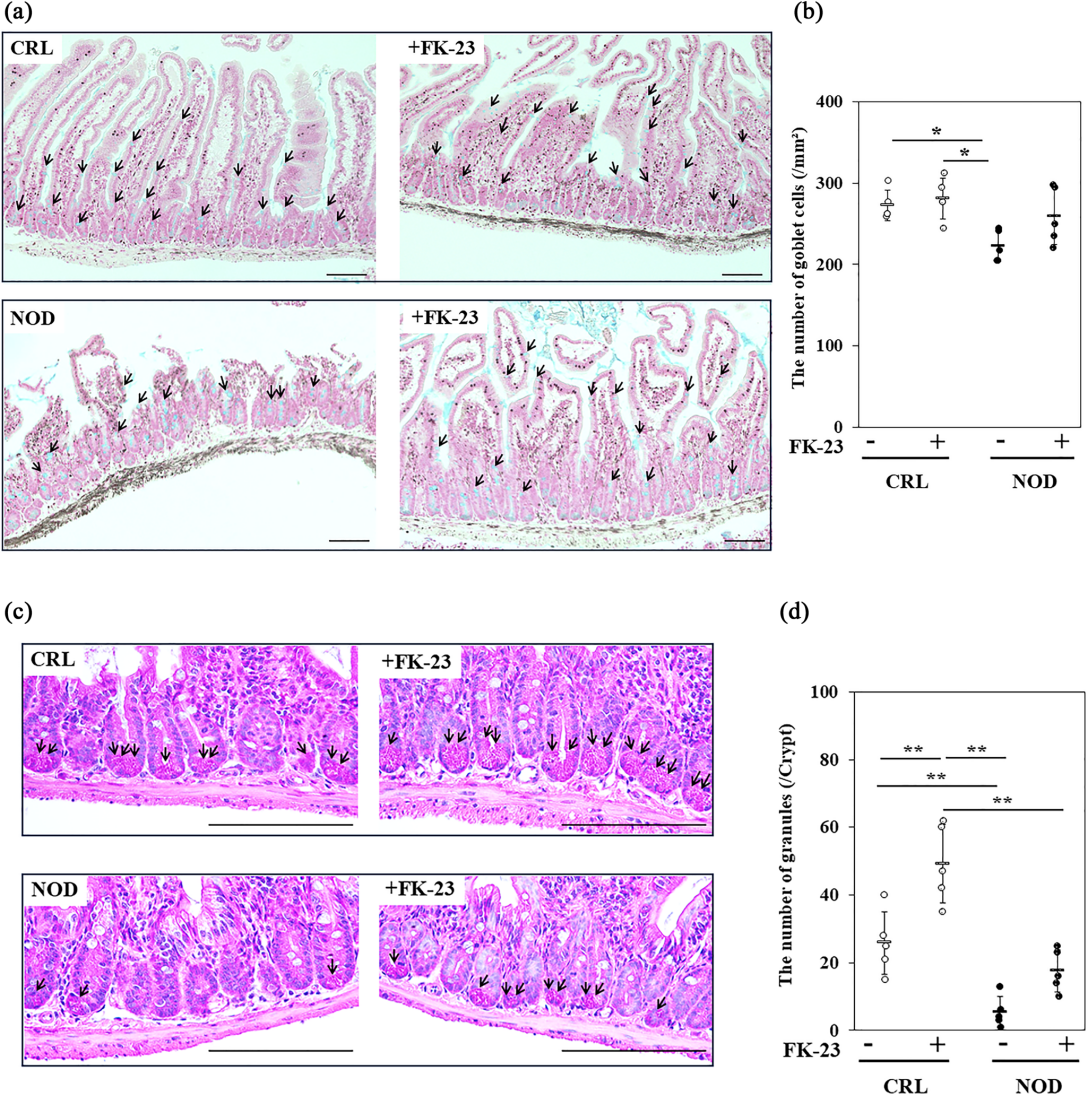
Small intestinal signs in 6‐week‐old NOD mice and the influence of FK‐23. (a) Alcian Blue staining of small intestine specimens. Scale bars: 100 µm. Arrows show examples of goblet cells. (b) Number of goblet cells in small intestine specimens from CRL, CRL+FK‐23, NOD and NOD+FK‐23 mice. (c) Haematoxylin and Eosin staining of small intestine specimens. Scale bars: 100 µm. Arrows show examples of granules. (d) Number of granules in crypts of small intestine specimens from CRL, CRL+FK‐23, NOD and NOD+FK‐23 mice. Values are presented as the mean ± SD. Tukey's *post hoc* test was used for comparisons among groups (*n* = 5): ^*^ *P* < 0.05 and ^**^ *P* < 0.01. Abbreviations: CRL, control; FK‐23, *Enterococcus faecalis* FK‐23; NOD, non‐obese diabetic.

After 4 weeks of age, Alcian Blue staining revealed lower goblet cell expression in NOD mice than in CRL mice at 6 weeks of age (Figure 6a,b). Haematoxylin and Eosin staining also revealed lower granule expression in the small intestinal crypt (Figure 6c,d). Dietary FK‐23 led to a tendency towards improvement in the decrease in goblet cell (*P *= 0.140) and granule expression in the small intestinal crypt (*P *= 0.140) (Figure 6a–d).

#### 3.3.3 Fluorescence‐activated cell sorting

Similar to the strategy shown in Figure 3a,b, flow cytometry analysis was performed on the SILP and LG at 6 weeks of age. Flow cytometry analysis revealed that the small intestine of 6‐week‐old NOD mice showed low expression of CD90.2^+^ cells and low expression of ILC3. In contrast, the expression of ILC1 was high. The expression of CD90.2^−^ cells was higher than in CRL, and ILC2 was abundant. However, in the small intestines of NOD mice receiving dietary FK‐23, the expression of ILC3 and ILC1 showed a trend towards improvement, whereas ILC2 showed a worsening trend (Figure 7a). Furthermore, Th17 cells were lower in the small intestine of the NOD mice than in that of the CRL mice, but this tended to improve with FK‐23 (Figure 7b). The expression of α4β7 integrin in Th17 cells in the small intestine did not differ between NOD and CRL mice and was not altered by dietary FK‐23. In the LG of 6‐week‐old NOD mice, CD90.2^+^ cells were more abundant than CRL cells, with no difference in ILC1 expression but more ILC3. The abundance of CD90.2^−^ cells and ILC2 was similar to that of 4‐week‐old mice, but ILCreg was more abundant. The NOD mice also had more NK cells than the CRL mice. [Correction added on 26 August 2026, after first online publication: The words “CRL” and “NOD” have been swapped in this sentence.] Dietary FK‐23 reduced the expression of several markers (ILC3, ILC2 and ILCreg) in CRL mice compared with untreated CRL mice. In contrast, dietary FK‐23 administration did not significantly alter ILC expression in NOD mice (Figure 7c). The number of Th17 cells in the LG of NOD mice was increased compared with that in CRL mice, but this showed a trend towards suppression with dietary FK‐23 administration (*P* = 0.085). Furthermore, the expression of α4β7 integrin in Th17 cells in the LG of 6‐week‐old NOD mice was reduced compared with that in CRL mice, but this showed a trend towards improvement with dietary FK‐23 administration (*P* = 0.145) (Figure 7d).

FIGURE 7Flow cytometry analysis of small intestinal lamina propria and lacrimal glands of 6‐week‐old NOD mice and the influence of FK‐23. (a) ILCs in the SILP. (b) Th17 cells in the SILP. (c) ILCs in the LG. (d) Th17 in the LG. Values are presented as the mean ± SD. Tukey's *post hoc* test was used for comparisons among groups (*n* = 5): ^*^ *P* < 0.05 and ^**^ *P* < 0.01. Abbreviations: CRL, control; FK‐23, *Enterococcus faecalis* FK‐23; ILCs, innate lymphoid cells; LG, lacrimal gland; NOD, non‐obese diabetic; SILP, small intestinal lamina propria; Th17, T helper 17.

**Figure:**
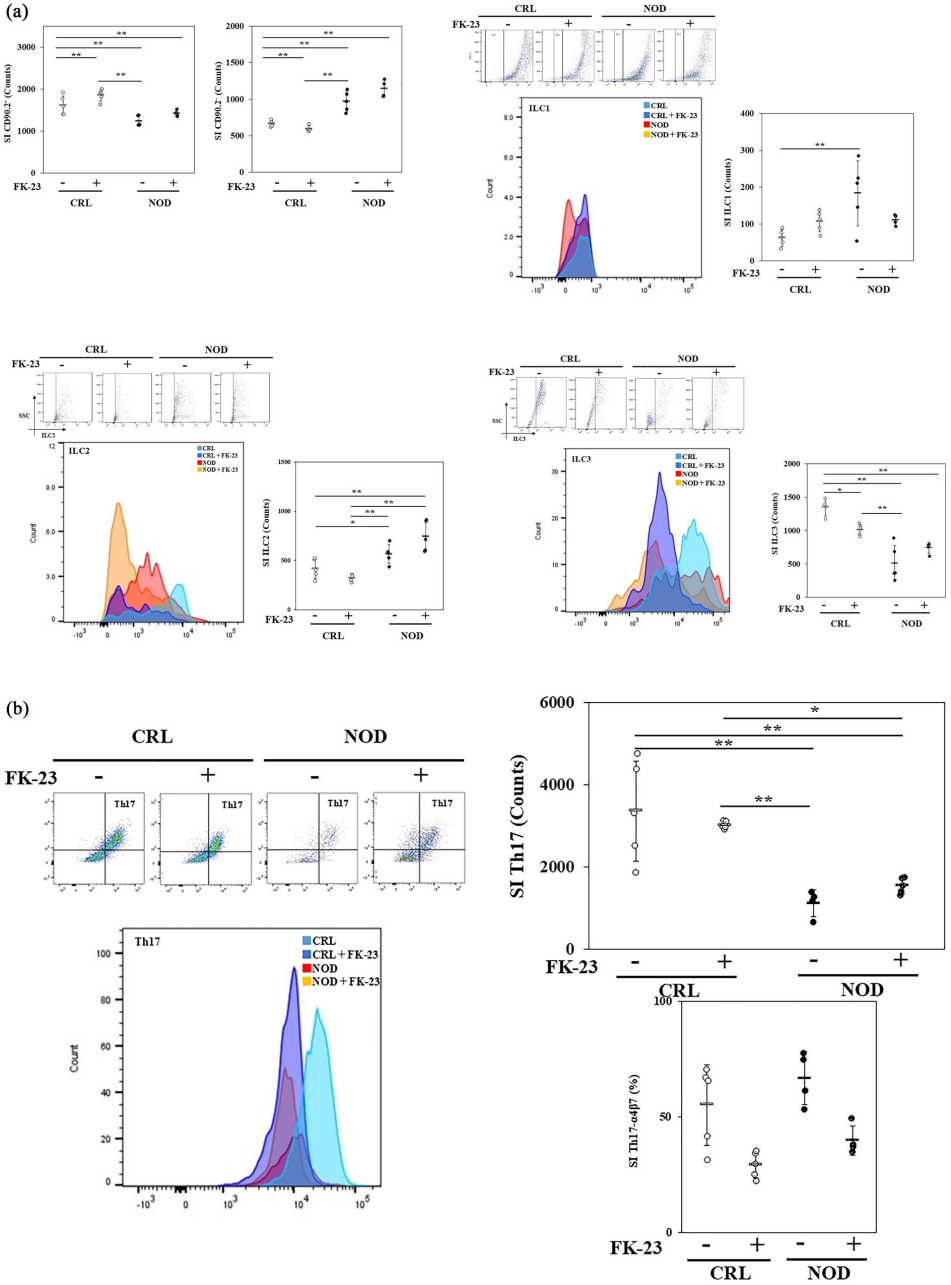

**Figure:**
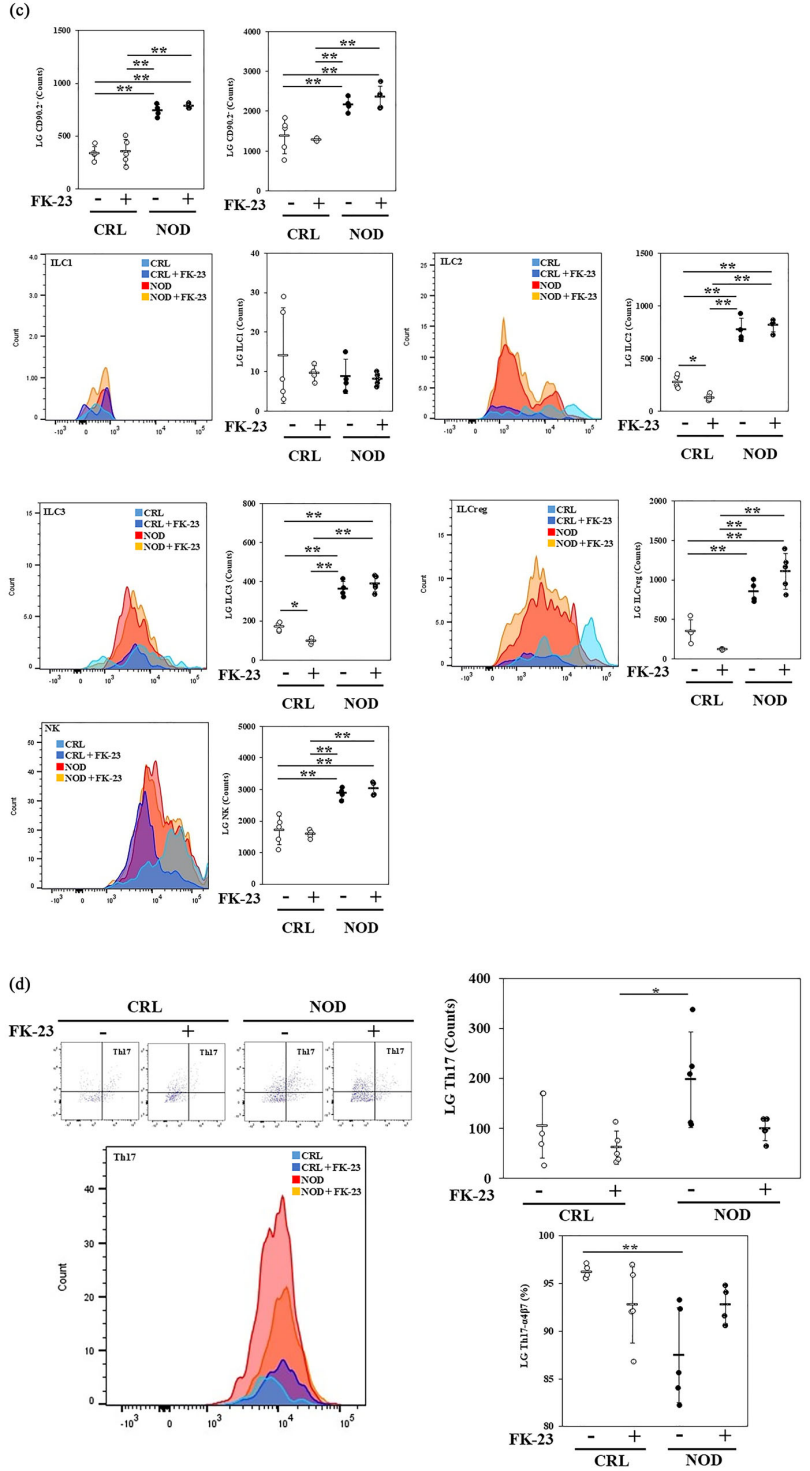

## 4 DISCUSSION

In this study, we used the NOD mouse model, which spontaneously develops SjD‐like dry eye disease, to investigate pathological changes in the LG and small intestine at 4 weeks of age, a stage considered preclinical, and at 6 weeks of age, with disease onset. Furthermore, we examined whether dietary FK‐23 from 4 weeks of age for 2 weeks could suppress disease development at 6 weeks of age.

The intestinal tract is constantly exposed to pathogenic microorganisms, such as enteropathogenic *Escherichia coli* and noroviruses, in addition to commensal microorganisms, including lactic acid bacteria and resident *E. coli*. To cope with this microbial burden, the host has evolved a highly sophisticated, multilayered defence system. Notably, crosstalk between the host and microbiota is essential for maintaining intestinal homeostasis, with the epithelial cell layer and immune system forming the core of this interaction (Round & Mazmanian, 2019; Allaire et al., 2019). The intestinal epithelium consists not only of absorptive enterocytes but also of goblet cells producing mucus, Paneth cells secreting antimicrobial peptides, tuft cells involved in anti‐helminth immunity, M cells specialized for antigen uptake, and enteroendocrine cells producing gastrointestinal hormones. These cells differentiate from epithelial stem cells located in the crypts and function as frontline physical barriers while exerting chemical and immunological roles through the secretion of mucus, antimicrobial peptides and hormones (Gieryńska et al., 2022; Kayama & Takeda, 2024; Okumura & Takeda, 2018). Paneth cells, in particular, contribute to the epithelial stem cell niche by secreting growth factors, such as EGF, Dll4 and Wnt3, thereby supporting epithelial development and maintenance (Sato et al., 2011).

Primary Sjögren's syndrome (pSS) is a systemic autoimmune disease with a worldwide prevalence of 0.01%–0.09%, characterized by leucocyte infiltration into exocrine glands, especially the salivary glands and LG, resulting in dry eye and dry mouth (Fox, 2005; Wu et al., 2023). Dry eye associated with pSS is clinically and pathophysiologically distinct from non‐Sjögren's dry eye, because immunologically mediated glandular damage leads to aqueous tear deficiency (Caban et al., 2022). Although the aetiology and pathogenesis of pSS remain poorly understood, increasing evidence implicates gut microbiota dysbiosis in the development of autoimmune diseases such as inflammatory bowel disease, systemic lupus erythematosus and rheumatoid arthritis (Chen et al., 2016; Halfvarson et al., 2017; Hevia et al., 2014). Dysbiosis has also been reported in pSS patients and is correlated with disease severity and gastrointestinal inflammation (de Paiva et al., 2016; Mandl et al., 2017).

These findings demonstrate that in 6‐week‐old NOD mice, not only LG pathology but also reduced goblet cell numbers and crypt granule expression were evident in the small intestine. Notably, similar intestinal abnormalities were already present at 4 weeks of age, prior to overt LG inflammation. These observations suggest that impaired intestinal function might precede and contribute to the development of LG inflammation in NOD mice.

ILCs play a crucial role not only in host defence against pathogens but also in autoimmune inflammation, tissue remodelling and homeostatic regulation. They respond to microbial and environmental cues and interact with T cells to maintain tissue homeostasis, and in inflammatory or immunodeficient conditions they can promote pathogenic immune responses (Klose & Artis, 2016; Sonnenberg & Hepworth, 2019). In the small intestine of NOD mice, the reduced frequency of ILC3s was partly restored by FK‐23 feeding, accompanied by improved villus morphology, suggesting a role for ILC3s in maintaining intestinal homeostasis. Moreover, the elevated frequency of ILC1s in NOD mice was attenuated by FK‐23, indicating that FK‐23 might suppress ILC1‐mediated small intestine tissue damage. It has been reported that the ILC3 subset is increased in the salivary glands of patients with pSS (Kawka et al., 2023). However, the results showed that although some subsets (ILC2) were highly expressed in the LGs of NOD mice at 4 weeks of age, the expression of most ILCs was significantly increased by 6 weeks of age. In contrast, although dietary FK‐23 suppressed symptoms in the LG, there was no change in LG expression of ILCs. This suggests that ILCs in the LG might contribute to the onset of inflammation but might also sense some kind of stress or homeostatic disruption and contribute to preparing and adjusting the immune response. Furthermore, dietary FK‐23 did not suppress proliferation of ILCs in the LGs of 6‐week‐old NOD mice, but it did suppress immune cell infiltration and tended to attenuate the decrease in tear secretion, suggesting that ILCs in the LG might be important for the development of dacryoadenitis but not for its healing.

Th17 cells and their signature cytokine, IL‐17, are strongly implicated in pSS pathology in both patients and mouse models (Verstappen et al., 2018; Witas et al., 2020). Although intestinal Th17 cells contribute to host defence, they can acquire pathogenic functions in autoimmune diseases (Schnell et al., 2021). In this study, Th17 cells were reduced in the small intestine but were increased in the LG of NOD mice as early as 4 weeks of age. Increased intestinal ILC1s and IFN‐inducible gene expression suggest that IFN‐γ derived from ILC1s might damage intestinal tissue, promoting aberrant migration of Th17 cells to the LG. Over time, exposure to IFN‐γ and ILCs might further drive Th17 cells towards a pathogenic phenotype. Collectively, RNA‐seq data indicated that Paneth cell dysfunction compromises the mucosal barrier, leading to increased bacterial burden, recruitment of CD14^+^ macrophages and upregulation of IL‐1β, IL‐12 and IL‐18, which, in turn, activate T‐bet‐dependent Th1, ILC1 and NK cell responses. IFN‐γ produced by these cells might further exacerbate epithelial and Paneth cell damage, creating a vicious cycle. Meanwhile, impaired Th17 and ILC3 functions, associated with reduced MHC class II expression, might hinder tissue repair, allowing immune cells to migrate from the intestine to the LG and drive inflammation. In NOD mice, α4β7 integrin expression in Th17 cells was reduced compared with that in CRL mice, suggesting impaired homing of Th17 cells to the small intestine and possible redistribution towards lacrimal gland tissue (Figure 7d).

The protective effects of FK‐23 appear to result from preservation of Paneth and goblet cell function, leading to enhanced induction and activation of ILC3s and Th17 cells in the intestine, promotion of epithelial repair, increased mucus and antimicrobial peptide production, and restoration of barrier integrity. Furthermore, dietary FK‐23 showed a tendency to restore α4β7 integrin expression in Th17 cells. Consequently, aberrant Th17 migration to the LG and ILC‐mediated inflammation were suppressed, preventing LG pathology. It is possible that intestinal inflammation or tissue damage induces inflammatory cytokines and chemokines, such as IFN‐γ‐associated signalling pathways, thereby promoting adhesion molecule expression and lymphocyte infiltration into the LG (Chen et al., 2017). According to Kamioka et al. (2022), possible mechanisms include interactions with glycan‐related factors, such as α1,2‐fucose and fucosyltransferase‐2, or regulation of the microbiota–ILC3–IL‐22 signalling pathway, which promotes epithelial homeostasis. Previous studies (Moon et al., 2020; Yun et al., 2021) have suggested that probiotics might improve dry eye symptoms through modulation of the gut microbiota. In addition, alterations in gut microbiota have been reported in NOD mice, and FK‐23 has also been shown to modify gut microbiota composition in a rat model of kidney disease (Takemura et al., 2024). Therefore, FK‐23 administration might also influence gut microbiota in NOD mice. Further studies evaluating gut microbiota composition and intestinal epithelial niche signalling pathways will be necessary to clarify the mechanisms underlying the observed effects of FK‐23.

## 5 CONCLUSION

In summary, our study emphasizes that intestinal abnormalities are the cause of ocular symptoms in the development of dry eye symptoms, such as in SjD, and provides evidence that strategies for symptom improvement based on the gut–eye axis are extremely important.

## AUTHOR CONTRIBUTIONS

All authors conceived and designed research; Kenji G performed experiments and analyzed data. All authors interpreted results of experiments. Kenji G prepared figures and drafted the manuscript. All authors edited and revised the manuscript. All authors approved the final version of manuscript and agree to be accountable for all aspects of the work in ensuring that questions related to the accuracy or integrity of any part of the work are appropriately investigated and resolved. All persons designated as authors qualify for authorship, and all those who qualify for authorship are listed.

## CONFLICT OF INTEREST

The authors declare no conflicts of interest.

## GENERATIVE AI STATEMENT

No generative AI tools were used in the preparation of this manuscript.

## Data Availability

All data supporting the results are in the publication. All original data in this publication are available upon reasonable request to the corresponding authors.
